# Mechanical Alloying: A Novel Technique to Synthesize Advanced Materials

**DOI:** 10.34133/2019/4219812

**Published:** 2019-05-30

**Authors:** Challapalli Suryanarayana

**Affiliations:** Department of Mechanical and Aerospace Engineering, University of Central Florida, Orlando, FL 32816-2450, USA

## Abstract

Mechanical alloying is a solid-state powder processing technique that involves repeated cold welding, fracturing, and rewelding of powder particles in a high-energy ball mill. Originally developed about 50 years ago to produce oxide-dispersion-strengthened Ni- and Fe-based superalloys for aerospace and high temperature applications, it is now recognized as an important technique to synthesize metastable and advanced materials with a high potential for widespread applications. The metastable materials produced include supersaturated solid solutions, intermediate phases, quasicrystalline phases, amorphous alloys, and high-entropy alloys. Additionally, nanocrystalline phases have been produced in virtually every alloy system. Because of the fineness of the powders, their consolidation to full density without any porosity being present is a challenging problem. Several novel methods have been developed to overcome this issue. Powder contamination during milling and subsequent consolidation constitutes another issue; this can be resolved, though expensive. A number of applications have been developed for these novel materials. This review article presents an overview of the process of mechanical alloying, mechanism of grain refinement to nanometer levels, and preparation of materials such as nanocomposites and metallic glasses. The application of mechanical alloying to synthesize some advanced materials such as pure metals and alloys, hydrogen storage materials, and energy materials is described. The article concludes with an outlook on future prospects of this technique.

## 1. Introduction

Advanced materials have been defined as those where first consideration is given to the systematic synthesis and control of the crystal structure and microstructure of materials to provide precisely tailored properties for demanding applications [1]. Even though different materials have been in use for several millennia, with rapid advances taking place in the technology sectors, there has been a great demand for materials that are stronger, stiffer, lighter, corrosion-resistant, and usable at higher temperatures than the existing and available materials. Thus, the high-tech industries have given an immense fillip to efforts in developing novel materials that will perform better for demanding applications. In response to these demands, materials scientists have been constantly on the lookout for novel methods to produce such materials.

**Figure 1:**
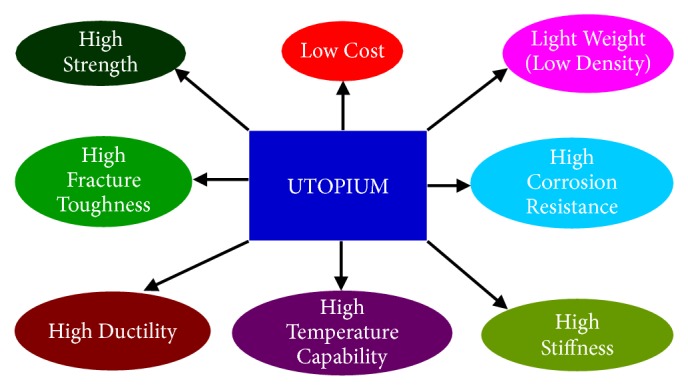
Typical properties expected of an ideal metal for present-day industrial applications include high strength, good ductility, excellent corrosion resistance, high fracture toughness, and the ability to be used at elevated temperatures. Since such a metal currently does not exist, let us call it* utopium* (not utopia, since the names of most metals end with ….um, e.g., aluminum, magnesium, titanium, uranium).

An ideal and most useful material should have an excellent combination of properties. It should have a high strength to withstand heavy loads and good ductility to be easily deformed into different shapes. But, traditional wisdom tells us that these two properties work in opposite directions; the ductility decreases when the strength increases. Additionally, the material should have good stiffness so that it does not deflect too much under load and high fracture toughness to resist easy and quick fracture. It will be most convenient if it is made up of light metals, when it can be used more effectively in the aerospace industry. Further, we also wish to have good corrosion resistance. Added to this excellent combination of properties, we would like the material to be easily available (to avoid geopolitical conflicts) and also inexpensive. Such a material does not exist at the moment and therefore let us call that imaginary ideal metal* utopium* (Figure 1). This will be like utopia, the perfect world. But, since the names of most metals end with “….um”, like aluminum, magnesium, titanium, uranium, etc., let us call that perfect (and elusive) metal, utopium.

It has long been realized that the material properties in general and, more importantly, the mechanical properties can be improved only by processing them under far-from equilibrium (or nonequilibrium) conditions [2]. Novel materials such as metallic glasses [3, 4], quasicrystalline alloys [5, 6], nanostructured materials [7, 8], high-entropy alloys [9], high-temperature superconductors, superhard carbo-nitrides, and thin-film diamond materials were unheard of prior to 1960s. Synthesis of martensite by quenching of steel to room temperature from an elevated temperature, where it exists in the austenitic condition, is an accepted method to strengthen steel. The mechanical properties can be further modified through tempering by reheating the martensitic steel to higher temperatures. Development of aircraft aluminum alloys through precipitation hardening in the 1910s–1930s laid the foundation for the development of high strength aluminum alloys. These principles are now further exploited to increase the strength of usually soft aluminum and magnesium alloys. Development of rapid solidification processing methods to achieve high solidification rates (about 10^6^ K/s) produced fine-grained alloys and metallic glasses with high mechanical strength, attractive soft magnetic properties, and vastly improved corrosion resistance. Synthesis of bulk metallic glasses at much slower solidification rates (<about 10^2^ K/s) and with larger section thicknesses (presently one can produce 80-mm diameter rods of fully glassy alloys) provided a much wider range of glassy materials. The appearance of nanostructured materials and high-entropy alloys on the scene has significantly enhanced the range of materials available to the materials scientists. In most of these cases, the academic curiosity of motivated researchers resulted in the synthesis of novel materials, some of which have subsequently found commercial applications.

Some of these “nonequilibrium processing” techniques include rapid solidification from the melt, mechanical alloying, laser processing, plasma processing, spray forming, physical and chemical vapor deposition techniques, and ion mixing. The relative advantages and disadvantages of these techniques have been discussed in a relatively recent publication [2]. However, in the present article, we will focus on the technique of mechanical alloying, describe the technique and its potential in synthesizing and developing advanced materials, and explore and exploit them for commercial applications.

## 2. The Process of Mechanical Alloying

Nonequilibrium processing of materials starts with energizing the material and using that excited (high-energy) state to process the materials to obtain the desired microstructure and properties. Researchers have been traditionally using temperature, pressure, light, or electricity to transform the equilibrium material into an energized condition. But, an easy and unconventional method to energize a material is to use “brute force” (or mechanical stress). The so-called severe plastic deformation processes (note that this name and the different variants under this category have come around much later than the technique of mechanical alloying) subject the material to high impact, shear, and torsional forces to energize the material.

The technique of mechanical alloying (MA) was developed in the 1960s in response to an industrial necessity. Faced with the question of identifying a material that has adequate strength both at intermediate and elevated temperatures, John Benjamin of INCO developed this method to uniformly disperse fine yttria (Y_2_O_3_) particles in a complex nickel-based alloy matrix [10]. Such alloys are now being referred to as oxide-dispersion strengthened (ODS) superalloys [11–14]. The “science” of the MA technique was developed much later in the late 1980s and is continuing to be expanded further.

MA involves loading of the individual elemental powders or prealloyed powders along with the grinding medium in a high-energy ball mill, typically maintaining a ball-to-powder weight ratio of 10:1 or higher. The process involves repeated cold welding, fracturing, and rewelding of powder particles. During this process the resulting powder size can be controlled by balancing the fracturing and welding events. About 1-2 wt.% of a process control agent (PCA) is used, especially when ductile metals are fabricated. The PCA is adsorbed on the surfaces of the powder particles and minimizes excessive cold welding among themselves and/or to the milling container and the grinding medium; this inhibits agglomeration of powder particles. The particle and grain sizes decrease progressively with milling time, reaching nanometer levels.

The process of MA can be carried out in small capacity and high-energy mills (SPEX mills) to produce about 10–20 g per run for alloy screening purposes, in medium-energy planetary mills to produce about 200–500 g at a time, or in low-energy much larger mills (attritors) to produce kg quantities. Currently, attritors capable of producing about 40–50 kg of powder in 24–48 hours are available in Russia for large-scale commercial applications. High-energy and high-speed mills that can produce about 5,000 kg of powder per hour are also available. In recent years, cryogenic milling (milling at liquid nitrogen temperatures) has become a popular method [15]. Facilities to cool the powders to low temperatures or heat them to high temperatures and monitor the pressure and temperature during milling are some of the attachments that are currently available.

The milling time decreases with an increase in the energy of the mill. As a rule of thumb, it can be estimated that a process that takes only a few minutes in the SPEX mill may take hours in an attritor and a few days in a commercial low-energy mill even though the actual details can be different depending on the efficiency of the different mills and the powder characteristics. Full details of the process including the effect of different variables on the alloying behavior may be found in two recent references [16, 17].

### 2.1. Mechanism of Alloying

**Figure 2:**
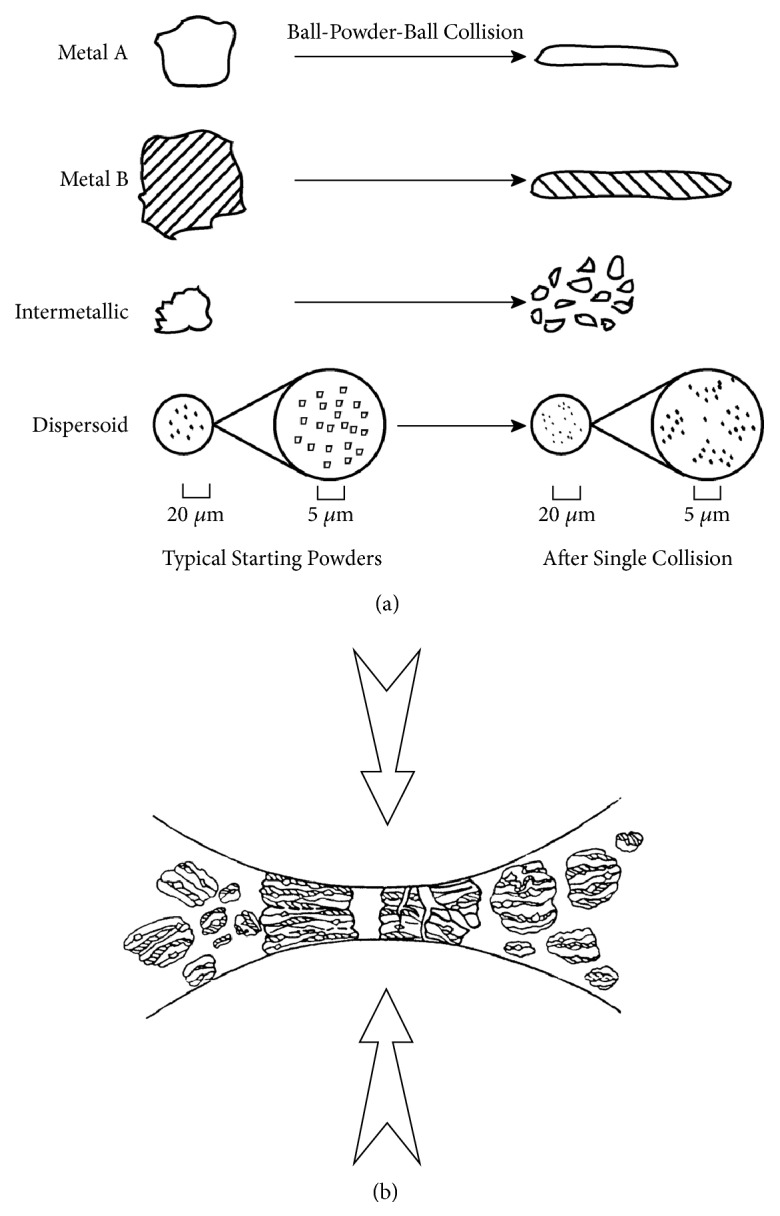
(a). Deformation characteristics of representative constituents of starting powders in mechanical alloying. Note that the ductile metal powders (metals A and B) get flattened, while the brittle intermetallic and dispersoid particles get fragmented into smaller particles. (b). Ball-powder-ball collision of powder mixture during mechanical alloying.

The effects of a single collision on each type of constituent powder particle are shown in Figure 2(a). The initial impact of the grinding ball causes the ductile metal powders to flatten and work harden. The severe plastic deformation increases the surface-to-volume ratio of the particles and ruptures the surface films of adsorbed contaminants. The brittle intermetallic powder particles get fractured and are refined in size. The oxide dispersoid particles are comminuted more severely.

Whenever two grinding balls collide, a small amount of the powder being milled is trapped in between them. Typically, around 1000 particles with an aggregate weight of about 0.2 mg are trapped during each collision (Figure 2(b)). During this process, the powder morphology can be modified in two different ways, depending on whether one is dealing with ductile–ductile, ductile–brittle, or brittle–brittle powder combinations. If the starting powders are soft metal particles, the flattened layers overlap and form cold welds. This leads to formation of layered composite powder particles consisting of various combinations of the starting ingredients. The more brittle constituents tend to become occluded by the ductile constituents and trapped in the composite. The work-hardened elemental or composite powder particles may fracture at the same time. These competing events of cold welding (with plastic deformation and agglomeration) and fracturing (with size reduction) continue repeatedly throughout the milling period. Eventually, at the steady-state condition, a refined and homogenized microstructure is obtained and the composition of the powder particles is the same as the proportion of the starting constituent powders.

Along with the cold welding event described above, some powder may also coat the grinding medium and/or the inner walls of the container. A thin layer of the coating is beneficial in preventing wear and tear of the grinding medium and also in preventing contamination of the milled powder with the debris. But, a too thick layer will result in lower yield and also possible compositional inhomogeneity of the powder, which should be avoided.

The generally accepted explanation for alloying to occur from blended elemental powders and formation of different types of phases is that a very fine and intimate mixture of the components (often lamellar if the constituent elements are sufficiently ductile) is formed after milling, if not the final product. Alloy formation is facilitated through crystalline defects (grain boundaries, dislocations, stacking faults, vacancies, and others) introduced into the material due to the intense cold working operation, which act as fast diffusion paths, and a slight rise in the powder temperature during milling, as a result of frictional forces and impact of the grinding balls against other balls and the surfaces of the container. If the final desired phase had not been formed directly by MA, then a short annealing treatment at an elevated temperature was found to promote diffusion and consequently alloy formation. Accordingly, it was shown that, by a proper choice of the process parameters during MA and by choosing an appropriate alloy composition, it is possible to produce a variety of alloys starting from metal powders. The thermodynamic criterion that the phase with the lowest free energy, under the continuous deformation conditions, would be the most “stable” phase has been found to be valid. A significant attribute of MA has been the ability to alloy metals even with positive heats of mixing that are difficult or impossible to alloy otherwise [18, 19].

The effects of MA are generally categorized under two groups:

(1)* Constitutional Changes*: Milling of the blended elemental powders could lead to the formation of solid solutions (both equilibrium and supersaturated), intermetallic phases (equilibrium, metastable (high temperature and/or high pressure), and quasicrystalline), and amorphous phases in alloy systems. The actual type of phase formed could be different depending on the alloy system, its composition, and the milling conditions employed. A number of metastable alloys have been produced this way.

(2)* Microstructural Changes*: As a result of MA, the processed materials could develop ultrafine-grained and nanostructured phases. In addition, uniform dispersion of a large volume fraction of very fine oxide or other ceramic particles can be achieved in different matrices which is either impossible or difficult by other processes. These novel nanocomposites can exhibit properties far superior to those that have conventional grain sizes and sometimes even completely new behavior [16, 17, 20].

In many cases, chemical reactions have also been found to occur leading to the production of pure metals from their ores, synthesis of alloys, and formation of novel materials and microstructures. Such a process has been referred to as mechanochemical processing [21]. All these effects have been well documented in the literature, and Table 1 summarizes the important attributes of MA.

Before discussing the synthesis of advanced materials and their applications, let us first describe the constitutional changes that can take place in MA materials.

## 3. Constitutional Changes

The literature on MA has several examples of the different equilibrium and nonequilibrium phases produced in different alloy systems. An exhaustive list of the results (up to date at the time of compilation) is available in [16, 17], and the list is constantly growing. We will, however, briefly highlight here some of the more recent and salient features in the following paragraphs.

### 3.1. Solid Solutions

Solid solution alloys exhibit high strength, changes in density, and suppression or elimination of undesirable second phases. Increasing the strength of alloys through precipitation of a second phase from a supersaturated solid solution during aging (precipitation hardening) has been known to materials scientists for over a hundred years. Further, since the strengthening effect is related to the size and volume fraction of the second phase particles and since both of them could be controlled by the extent of supersaturation and the aging parameters, achievement of extended solid solubility limits in alloy systems is very desirable. Solid solutions—both equilibrium and supersaturated—are still being produced [22–24].

Rules similar to those developed by Hume-Rothery for solid solution formation in binary noble-metal alloy systems under equilibrium conditions also seem to be generally applicable to supersaturated solutions obtained by MA, even though exceptions have been noted in some cases [25, 26].

As will be described later, the mechanically alloyed materials exhibit very fine grain sizes, often reaching nanometer levels. Since these nanocrystalline materials contain a large volume fraction of atoms in the grain boundaries, the diffusivity of solute atoms is significantly enhanced [27], and this would lead to increases in the solid solubility levels. As a general rule of thumb, solute elements which exhibit limited solubility under equilibrium conditions exhibit high solubility levels under the nonequilibrium conditions of MA. Even though solid solubility extensions have been achieved by other nonequilibrium processing techniques as well (e.g., rapid solidification processing, vapor deposition, and laser processing of materials), there appear to be differences in the solid solubility extensions achieved by MA and other methods [26]. An important observation made in MA materials is that the solute atoms segregate to grain boundaries from where they could diffuse into grains and form solid solutions.

### 3.2. Intermetallic Phases

Intermetallic phases (or intermetallics, for short) are typically ordered compounds existing at simple ratios of the two or more elements involved. Because of their ordered nature, they are strong and hard, stiff, have high melting points, and also exhibit other interesting behaviors such as excellent corrosion/oxidation resistance. However, they usually suffer from low ambient temperature ductility, again due to the ordered nature of the compounds. A new family of intermetallics showing high ductility at room temperature, and based on rare-earth elements, has been recently developed [28, 29]. Since MA is known to (a) disorder the ordered lattices, (b) refine grain sizes down to nanometer levels, and (c) also alter the crystal structures, all these effects could induce some amount of plasticity into the material. In fact, the potential to ductilize the normally brittle intermetallics has been the driver for accelerated research into the MA of intermetallics.

A number of intermetallics (both equilibrium and metastable) and high-temperature or high-pressure phases have been synthesized by MA starting from elemental powder blends [16, 17, 24, 30]. More interestingly, quasicrystalline phases with the forbidden crystal symmetries, first reported in rapidly solidified Al-Mn alloys, have also been synthesized by MA in a number of alloy systems [5, 31]. Applications for these exotic materials have been limited. Based on their high hardness, low friction, and low surface reactivity, quasicrystalline materials have been used to coat nonstick frying pans. Also, steels reinforced with small quasicrystalline particles are very strong and have been used for acupuncture and surgery, dental instruments, and razor blades [32].

The most significant change that occurs in MA materials is the formation of amorphous phases and this will be discussed in the next section.

## 4. Metallic Glasses

Amorphous alloys are materials that lack crystallinity and have a random arrangement of atoms. Because of this extreme disorder, these materials exhibit a very interesting combination of high strength, good corrosion resistance, and useful electrical and magnetic properties. These novel materials have been traditionally produced starting from the vapor or liquid states. Amorphous or noncrystalline alloys based on metals are commonly referred to as metallic glasses. At present, there is worldwide activity in the area of metallic glasses and bulk metallic glasses [4, 33].

Metallic glasses were first produced by rapid solidification processing (RSP) in 1960 in the form of thin ribbons by solidifying the alloy melt at rates >10^6^ K/s [34]. Subsequent developments include the synthesis of bulk metallic glasses with a large section thickness of a few centimeters. This is possible in multicomponent alloys which could be solidified into the glassy condition at low critical cooling rates of <10^2^ K/s [4]. The current record is an 80-mm diameter glassy rod produced by solidification in a Pd_42.5_Cu_30_Ni_7.5_P_20_ alloy (the subscripts represent the atomic percentage of the elements in the alloy) [35]. While solidification methods produce the metallic glasses either in thin ribbon or bulk form directly from the liquid, the amorphous phases are in powder form by MA methods. These need to be consolidated to bulk shape for subsequent characterization and applications.

Amorphous alloys were synthesized by MA first in an Y-Co intermetallic compound [36] and later in a blended elemental Ni-Nb powder mixture [37]. Amorphous phases have now been synthesized in a very large number of alloy systems starting from either blended elemental powder mixtures, intermetallic compounds, or mixtures of them [38]. Comparisons have been frequently made between the amorphous alloys produced by MA and RSP techniques, and both similarities and differences have been noted [26]. Further, the mechanism of glass formation appears to be significantly different depending upon whether it is produced by MA or RSP methods.

Several different criteria have been proposed to explain the glass-forming ability of alloys produced by solidification methods. These include the reduced glass transition temperature, *T*_rg_ = *T*_g_/*T*_l_, where *T*_g_ and *T*_l_ represent the glass transition and liquidus temperature of the alloy, respectively; presence of deep eutectics in the phase diagrams; and significant differences in the atomic sizes of the constituent elements, leading to development of strain energy in the solid solution. When the strain energy exceeds a critical value, determined by the size and amount of the solute atoms, it destabilizes the crystal lattice and results in the formation of an amorphous phase. With the introduction of bulk metallic glasses nearly thirty years ago, a very large number of new criteria, mostly based on the glass transformation temperatures (*T*_g_, *T*_x_, and *T*_l_, where *T*_x_ represents the crystallization temperature of the amorphous alloy), have been proposed. In spite of a very large number of criteria proposed [4], it has not been possible yet to accurately predict the glass-forming ability of alloys [39].

There are some significant differences in the formation of metallic glasses by the MA and RSP methods. For example, metallic glasses are obtained by MA in almost every alloy system provided that sufficient energy has been stored in the powder. But, metallic glasses are obtained by RSP methods only in certain composition ranges—about 20 at.% metalloid content in metal-metalloid systems and different compositions in metal-metal systems, mostly at compositions corresponding to deep eutectics. Additionally, glass formation by MA occurs by the accumulation of crystal defects, which raises the free energy of the crystalline phase above that of the hypothetical glassy phase, under which conditions the crystalline phase becomes destabilized. On the other hand, for glass formation by the RSP methods, the critical cooling rate for glass formation needs to be exceeded so that formation of the crystalline nuclei is completely suppressed. Since MA is a completely solid-state powder processing technique, the criteria for amorphous phase formation should be different from those of solidification methods. A new criterion was proposed; that is, the number of intermetallics present in the constituent phase diagrams determines the glass-forming ability of alloys processed by MA—the more the number of intermetallics in the constituent phase diagrams, the easier it is to amorphize the powder blend [40].

Amorphization by MA occurs when the free energy of the hypothetical amorphous phase, *G*_A_, is lower than that of the crystalline phase,* G*_C_; i.e., *G*_A_ < *G*_C_. A crystalline phase normally has a lower free energy than the amorphous phase. But, its free energy can be increased by introducing a variety of crystal defects such as dislocations, grain boundaries, and stacking faults. If an intermetallic has formed, then additional energy can be introduced by disordering the crystal lattice. By this approach, it is then possible to obtain a situation when

(1) $$\begin{array}{c} G_{A}<G_{C}+G_{D} \end{array}$$

where *G*_D_ is the free energy increase due to introduction of crystal defects. It has been shown that the presence of intermetallics in an alloy system can significantly increase the energy contributing to amorphization. This is due to two important effects. Firstly, disordering of intermetallics contributes an energy of about 15 kJ/mol to the system. Secondly, a slight change in the stoichiometry of the intermetallic increases the free energy of the system drastically. Additionally, grain size reduction contributes about 5 kJ/mol. Further, disordering of intermetallics has also been shown to be possible by heavy deformation. Since MA reduces the grain size to nanometer levels and also disorders the usually ordered intermetallics, the energy of the milled powders is significantly raised. In fact, it is raised to a level above that of the hypothetical amorphous phase, and thus formation of the amorphous phase is favored over the crystalline phase.

### 4.1. Mechanical Crystallization

It was reported that an amorphous phase, formed by milling, transforms into a crystalline phase on continued milling and that this phenomenon has been referred to as mechanical crystallization [41, 42]. The process of mechanical crystallization is very unusual and offers many opportunities to design novel alloys with improved properties. For example, it will be possible to produce the alloy in either a fully amorphous, amorphous + nanocrystalline composite, or a completely crystalline state with different grain sizes, depending upon the time of milling or the temperature and time of subsequent annealing of the amorphous alloy.

**Figure 3:**
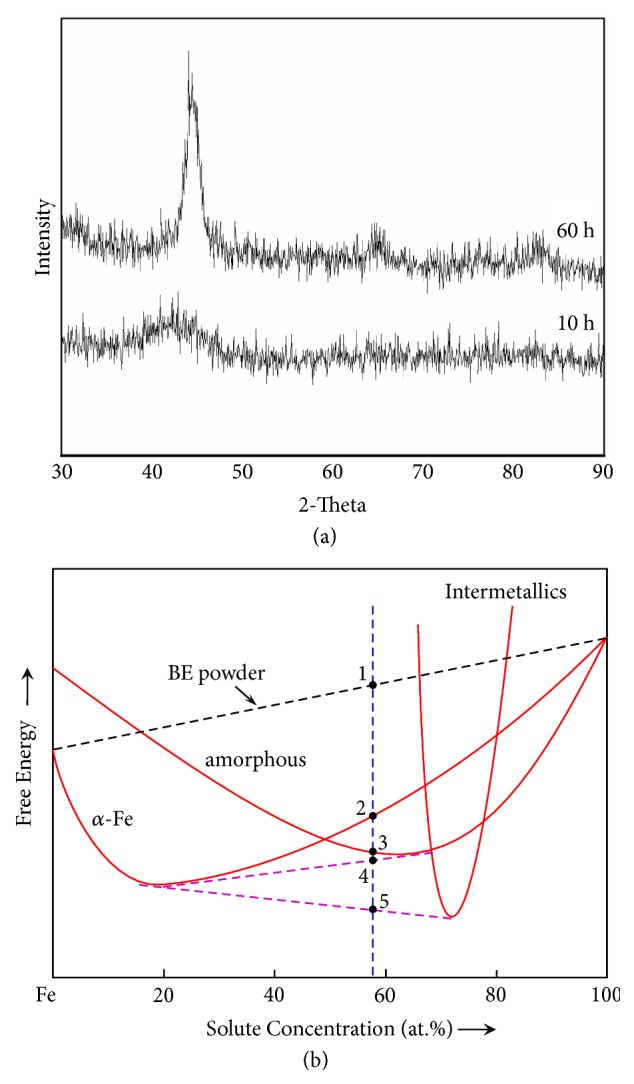
(a). X-ray diffraction patterns of the Fe_42_Ge_28_Zr_10_B_20_ powder blend milled for 10 and 60 h. While the powder milled for 10 h shows the presence of an amorphous phase, the powder milled for 60 h shows the presence of a crystalline phase coexisting with the amorphous phase suggesting that the amorphous phase has crystallized. (b) Hypothetical free energy vs. composition diagram to explain the mechanism of mechanical crystallization in the Fe_42_X_28_Zr_10_B_20_ system. Note that point “1” represents the free energy of the blended elemental powders. Similarly, point “2” represents formation of the *α*-Fe solid solution containing all the alloying elements in Fe, point “3” formation of the homogeneous amorphous phase, point “4” a mixture of the amorphous phase with a different solute content (amorphous) from that at “3” and the solid solution *α*-Fe with different solute content, and point “5” the equilibrium situation when the solid solution and intermetallic phases coexist.

Figure 3(a) shows the XRD patterns of the blended elemental powder mixture of the composition Fe_42_Ge_28_Zr_10_B_20_ mechanically alloyed for 10 and 60 h [42]. While the pattern at 10 h of milling clearly shows the presence of an amorphous phase, the pattern at 60 h of milling shows the presence of a crystalline phase, superimposed over the broad and diffuse halo, representing the amorphous phase. The crystalline phase has been identified as the *α*-Fe solid solution containing the solute elements present in the blend. This *α*-Fe phase is the first phase to crystallize from the amorphous alloy (as in primary crystallization), and external annealing of the milled powder showed that the amount of the *α*-Fe increased along with the presence of other intermetallics. Eutectic crystallization (simultaneous formation of two crystalline phases) has also been observed in other alloy systems.

Mechanical crystallization has also been observed in several other alloy systems that formed the amorphous phase on MA. In fact, it is now becoming apparent that this phenomenon of mechanical crystallization is not as rare as it was once thought to be.

Many reasons have been suggested for the formation of a crystalline phase after the formation of an amorphous phase during MA. These include (a) rise in temperature to a level above that of the crystallization temperature of the amorphous alloy, (b) powder contamination due to which a stable crystalline impurity phase forms, (c) phenomenon of inverse melting, and (d) basic thermodynamic considerations. It now appears clear that the basic thermodynamic stabilities of the different phases under the different conditions of milling are responsible for mechanical crystallization. Such a situation can be explained with reference to a free energy versus composition diagram.

Figure 3(b) is a schematic diagram showing the variation of free energy with composition for the Fe_42_X_28_Zr_10_B_20_ (X = Al, Ge, and Ni) alloy systems investigated. The possible (stable and metastable) constitution in this system is (a) blended elemental (BE) powder mixture, (b) *α*-Fe, the solid solution of all the alloying elements in Fe, (c) an amorphous phase, (d) intermetallic phases, and (e) different combinations of these phases. The stability of any phase will be determined by its relative position in the free energy vs. composition plot—the lower the free energy, the more stable the phase is. Since it is possible to have a large number of intermetallic phases in this multicomponent system, and since it is difficult to indicate each of them separately, for simplicity, all of them are grouped together as “intermetallics”.

The free energy of the BE powder mixture is indicated by point “1”, which represents the point of intersection of the composition vertical with the line joining the free energies of pure Fe and the “solute”. On milling this powder, a solid solution phase containing all the solute elements in Fe (or a mixture of intermetallics and a solid solution, in some cases) is seen to form, and its free energy is indicated by point “2”. The solid solution forms because it has a lower free energy than the BE powder mixture. Since MA introduces a variety of crystal defects, the crystalline phases in the milled powders will contain excess energy. This energy will continue to increase with milling time and reach a value which is above that of the metastable amorphous phase. Thus, the amorphous phase gets stabilized (point “3”). On primary crystallization of the amorphous phase, the new constitution will be a mixture of the *α*-Fe solid solution (or intermetallics) and the amorphous phase, which now has a composition different from that of the original amorphous phase. Further, the new solid solution phase also has a composition different from the original *α*-Fe phase. The free energy of the mixture of this solid solution and the amorphous phase, indicated by “point 4”, will have a free energy lower than that of the amorphous phase. The equilibrium mixture of the *α*-Fe solid solution and “intermetallics” will have the lowest free energy of all the phase mixtures, as indicated by point “5” in the figure. The process of mechanical crystallization is unique to the MA process and can be achieved only on continued milling of the amorphous powder or milling of an amorphous alloy obtained by other methods.

## 5. Nanostructured Materials

Another important attribute of MA has been microstructural refinement. The most common observation is that grain refinement takes place resulting in the formation of very fine grains, often down to nanometer levels. A two-phase alloy under equilibrium conditions can transform to a single-phase alloy due to formation of a supersaturated solid solution. An amorphous alloy can crystallize under the action of severe plastic deformation. Most usefully, one can also introduce a high volume fraction of fine second phase particles and obtain a composite with a uniform distribution of the second phase particles. By a proper choice of the size of the matrix and/or the reinforcement phase, one can produce nanocomposites, a very fruitful area for investigations. In fact, MA has been the most popular technique to synthesize nanostructured materials and nanocomposites in a number of alloy systems.

It has been frequently demonstrated that mechanically alloyed materials show powder particle refinement as well as grain refinement as a function of the milling time. These sizes decrease very rapidly in the early stages of milling and more gradually later. Eventually, in almost all the cases, the grain size of the mechanically alloyed materials is in the nanometer region, unless the powder becomes amorphous. The minimum grain size achieved has been reported to be a few nanometers, ranging typically from about 5 to 50 nm, but depending on the material and processing conditions. For example, it was reported that the grain size decreases with increasing milling energy, higher ball-to-powder weight ratio, and lower temperatures. As is well known, nanostructured materials exhibit high strength, good ductility, excellent sintering characteristics, and interesting electrical and magnetic properties [7, 8, 27, 43]. The ease with which nanostructured materials can be synthesized is one reason why MA has been extensively employed to produce nanocrystalline and nanocomposite materials.

From high-resolution transmission electron microscopy (TEM) observations, it was noted that in the early stages of milling, due to the high deformation rates experienced by the powder, deformation was localized within shear bands [44]. These shear bands, which contain a high density of dislocations, have a typical width of approximately 0.5 to 1.0 *μ*m. Small grains, with a diameter of 8–12 nm, were seen within the shear bands, and electron diffraction patterns suggested significant preferred orientation. With continued milling, the average atomic level strain increased due to increasing dislocation density, and at a certain dislocation density within these heavily strained regions, the crystal disintegrated into subgrains that are separated by low-angle grain boundaries. This resulted in a decrease of the lattice strain. The subgrains formed this way were of nanometer dimensions and are often between 20 and 30 nm.

On further processing, deformation occurred in shear bands located in previously unstrained parts of the material. The grain size decreased steadily and the shear bands coalesced. The small-angle boundaries are replaced by higher angle grain boundaries, implying grain rotation, as reflected by the absence of texture in the electron diffraction patterns and random orientation of the grains observed from the lattice fringes in the high-resolution TEM micrographs. Consequently, dislocation-free nanocrystalline grains are formed. This is the currently accepted mechanism of nanocrystal formation in MA processed powders.

The grain size of the milled materials decreases with milling time and reaches a saturation level when a balance is established between the fracturing and cold welding events. This minimum grain size, *d*_min_, is different depending on the material and milling conditions. The value of *d*_min_ achievable by milling is determined by the competition between the plastic deformation via dislocation motion that tends to decrease the grain size and the recovery and recrystallization behavior of the material that tends to increase the grain size. This balance gives a lower bound for the grain size of pure metals and alloys.

**Figure 4:**
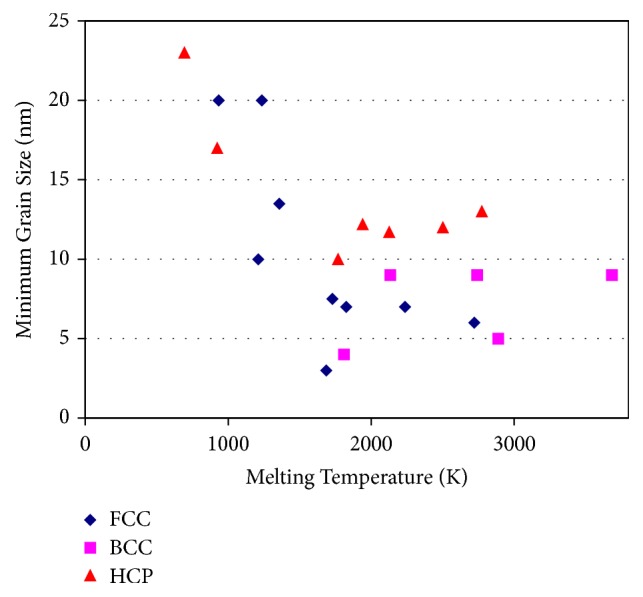
Variation of minimum grain size with temperature in mechanically milled FCC, HCP, and BCC metals.

The *d*_min_ obtained is different for different metals and is also found to vary with the crystal structure (Figure 4). In most of the metals, the minimum grain size attained is in the nanometer dimensions. But, metals with a body-centered cubic (BCC) crystal structure reach much smaller values in comparison to metals with the other crystal structures, related to the difficulty of extensive plastic deformation and consequent enhanced fracturing tendency during milling. Ceramics and intermetallic compounds are much harder and usually more brittle than the metals on which they are based. Therefore, intuitively, one expects that *d*_min_ of these compounds is smaller than those of the pure metals, but this does not appear to be the case always.

It was also reported that *d*_min_ decreases with an increase in the melting temperature of the metal [45]. As shown in Figure 4, this trend is amply clear in the case of metals with close-packed structures (face-centered cubic (FCC) and hexagonal close-packed (HCP)), but not so clear in metals with a BCC structure. Another point of interest is that the difference in grain size is much less among metals that have high melting temperatures; the minimum grain size is virtually constant. Thus, for the HCP metals Co, Ti, Zr, Hf, and Ru, the minimum grain size is almost the same even though the melting temperatures vary between 1495°C for Co and 2310°C for Ru.

Analysis of the existing data on *d*_min_ of mechanically milled pure metals shows that the normalized minimum grain size, *d*_min_/***b***, where  ***b*** is the Burgers vector of the dislocations, decreases with increasing melting temperature, activation energy for self-diffusion, hardness, stacking fault energy, bulk modulus, and the equilibrium distance between two edge dislocations. The *d*_min_ value is also affected by milling energy (the higher the milling energy, the smaller the grain size), milling temperature (the lower the milling temperature, the smaller the grain size), and alloying additions (the minimum grain size is smaller for solid solutions than for pure metals, since solid solutions are harder and stronger than the pure metals on which they are based).

It was suggested that *d*_min_ is determined by the minimum grain size that can sustain a dislocation pile-up within a grain and by the rate of recovery. Based on the dislocation pile-up model, the critical equilibrium distance between two edge dislocations in a pile-up,* L*_c_ (which could be assumed to be the crystallite or grain size in milled powders), was calculated [46] using the equation

(2) $$\begin{array}{c} L_{c}=\frac{3\;Gb}{\left(1-\nu \right)H} \end{array}$$

where* G* is the shear modulus, **b** is the Burgers vector, *ν* is the Poisson's ratio, and* H* is the hardness of the material. According to the above equation, increased hardness results in smaller values of *L*_c_ (grain size), and an approximate linear relationship was observed between *L*_c_ and the minimum grain size obtained by milling of a number of metals. Other attempts have also been made to theoretically predict *d*_min_ on the basis of thermodynamic properties of materials [47].

## 6. Nanocomposites

Achievement of a uniform distribution of the reinforcement in a matrix is essential to the achievement of good and uniform mechanical properties in composites. Further, the mechanical properties of the composite tend to improve with increasing volume fraction and/or decreasing particle size of the reinforcement [48]. Traditionally, a reasonably large volume fraction of the reinforcement could be added, if the size of the reinforcement is large (on a micrometer scale or larger). But, if the reinforcement size is very fine (of nanometer dimensions), then the volume fraction added is typically limited to about 2 to 4 %. However, if a large volume fraction of nanometer-sized reinforcement can be introduced, the mechanical properties of the composite are likely to be vastly improved. Therefore, several investigations have been undertaken to produce improved nanocomposites through MA [20].

Composites are traditionally produced by solidification processing methods since the processing is inexpensive and large tonnage quantities can also be produced. But, it is not easy to disperse fine reinforcement particles in a liquid metal since agglomeration of the fine particles occurs and clusters are produced. Further, wetting of the particles is poor, and consequently even the most vigorous stirring is not able to break the agglomerates. Additionally, the fine powders tend to float to the top of the melt during processing of the composites through solidification processing. Thus, solid-state processing methods such as MA are effective in dispersing fine particles (including nanoparticles). An added advantage is that, since no melting is involved, even a large volume fraction of the reinforcement can be introduced.

Some of the specific goals sought in processing of nanocomposite materials through MA include incorporation of a high volume percentage of ultrafine reinforcements and achievement of high ductility, and even superplasticity in some composites. It is also possible that, due to the increased ductility, the fracture toughness of these composites could be higher than that of their coarse-grained counterparts. In line with these goals, we have, in recent times, accomplished (a) achievement of a very uniform distribution of the reinforcement phases in different types of matrices, including dispersion of a high volume fraction of Al_2_O_3_ in Al [49] and of Ti_5_Si_3_ in *γ*–TiAl [50, 51], and (b) synthesis of MoSi_2_+Si_3_N_4_ composites for high-temperature applications [52], among others. However, we will describe here only the development of Ti_5_Si_3_+*γ*–TiAl composites, as a typical example.

Lightweight intermetallic alloys based on *γ*-TiAl are promising materials for high-temperature structural applications, e.g., in aircraft engines or stationary turbines. Even though they have many desirable properties such as high specific strength and modulus, both at room and elevated temperatures, and good corrosion and oxidation resistance, they suffer from inadequate room temperature ductility and insufficient creep resistance at elevated temperatures, especially between 800 and 850°C, an important requirement for elevated temperature applications of these materials. Therefore, current research programs have been addressing the development of high-temperature materials with adequate room temperature ductility for easy formability and ability to increase the high-temperature strength by a suitable heat treatment or alloying additions to obtain sufficient creep resistance at elevated temperatures.

**Figure 5:**
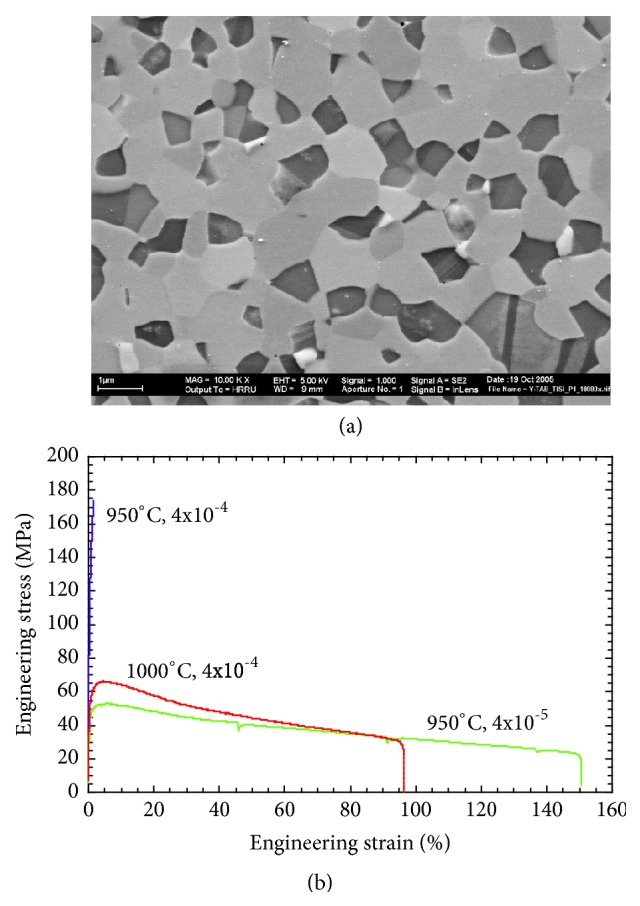
(a) Scanning electron micrograph of the *γ*-TiAl + 60 vol.% Ti_5_Si_3_ composite specimen showing that the two phases are very uniformly distributed in the microstructure. Such a microstructure is conducive to achieving superplastic deformation under appropriate conditions of testing. (b) Tensile stress-strain curve of *γ*-TiAl + 60 vol.% Ti_5_Si_3_ composite showing that superplastic deformation was achieved at 950°C with a strain rate of 4x10^−5^ s^−1^ and at 1000°C with a strain rate of 4x10^−4^ s^−1^.

Composites of *γ*-TiAl and *ξ*-Ti_5_Si_3_ phase, with the volume fractions of the *ξ*-Ti_5_Si_3_ phase varying from 0 to 60 vol.%, were produced by MA of a combination of the blended elemental and prealloyed intermetallic powders. Fully dense and porosity-free compacts were produced by hot isostatic pressing, with the resulting grain size of each of the phases being about 300-400 nm.  Figure 5(a) shows scanning electron micrographs of the *γ*-TiAl+60 vol.%  *ξ*-Ti_5_Si_3_ composite showing that the two phases are very uniformly distributed throughout the microstructure. Materials exhibiting such a microstructure (roughly equal amounts of the two phases, small grain sizes, and a uniform distribution of the two phases) are expected to be deformed superplastically. To test this hypothesis, both compression and tensile testing of these composite specimens were conducted at different temperatures and strain rates. From the tensile stress-strain plots of the *γ*-TiAl+60 vol.%  *ξ*-Ti_5_Si_3_ composite specimens shown in Figure 5(b), it is clear that the specimens tested at 950°C with a strain rate of 4×10^−5^ s^−1^ and at 1000°C with a strain rate of 4×10^−4^ s^−1^ exhibited large ductility of nearly 150 and 100%, respectively. Considering that this composite is based on a ceramic material (Ti_5_Si_3_), this is a very high amount of deformation, suggestive of superplastic deformation. Final proof is provided by TEM investigations that confirm the continued stability of the equiaxed microstructure after deformation.

## 7. Applications

**Figure 6:**
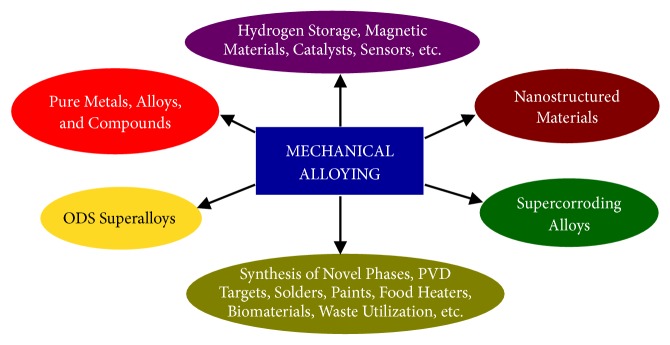
An overview of the applications of the MA process.

As has been frequently pointed out in the literature, the technique of MA was developed out of an industrial necessity in 1965 to produce ODS nickel-based superalloys. Since then a number of new applications have been explored for mechanically alloyed materials (Figure 6). Apart from the ODS alloys, which continue to be used in the high-temperature applications area, MA powders have also been used for other applications and newer applications are also being explored. These novel applications include direct synthesis of pure metals and alloys from ores and scrap materials, super-corroding alloys for release into ocean bottoms, development of PVD (physical vapor deposition) targets, solders, paints, etc., hydrogen storage materials, photovoltaic materials, catalysts, and sensors.

A novel and simple application of MA powders is in the development of meals, ready to eat (MRE). In this application, very finely ground Mg-based alloy powders are used. When water is added to these fine Mg-Fe powders, heat is generated according to the following reaction.

(3) $$\begin{array}{c} Mg\left(\;Fe\;\right)+2H_{2}O\to Mg{\left(\;OH\;\right)}_{2}+H_{2}+Heat \end{array}$$

This heat is utilized to warm the food in remote areas, where cooking or heating facilities may not be available, e.g., in war-torn areas. These “food heaters” were widely used during the Desert Storm operations in the 1990s. Let us now look at some of the other applications.

### 7.1. Pure Metals and Alloys

The technique of MA has been employed to produce pure metals and alloys from ores and other raw materials such as scrap. The process is based on chemical reactions induced by mechanical activation. Most of the reactions studied have been displacement reactions of the type

(4) $$\begin{array}{c} MO+R\to M+RO \end{array}$$

where a metal oxide (*MO*) is reduced by a more reactive metal (reductant,* R*) to the pure metal* M*. In addition to oxides, metal chlorides and sulfides have also been reduced to pure metals this way. Similar reactions have also been used to produce alloys and nanocomposites [21, 53].

All solid-state reactions involve the formation of a product phase at the interface of the component phases. Thus, in the above example, the metal* M* forms at the interface between the oxide* MO* and the reductant* R* and physically separates the reactants. Further growth of the product phase involves diffusion of atoms of the reactant phases through the product phase, which constitutes a barrier layer preventing further reaction from occurring. In other words, the reaction interface, defined as the nominal boundary surface between the reactants, continuously decreases during the course of the reaction. Consequently, kinetics of the reaction are slow and elevated temperatures are required to achieve reasonable reaction rates.

MA can provide the means to substantially increase the reaction kinetics of chemical reactions, in general, and the reduction reactions, in particular. This is because the repeated cold welding and fracturing of powder particles increase the area of contact between the reactant powder particles by bringing fresh surfaces to come into contact repeatedly due to a reduction in particle size during milling. This allows the reaction to proceed without the necessity for diffusion through the product layer. As a consequence, reactions that normally require high temperatures will occur at lower temperatures, or even without any externally applied heat. In addition, the high defect densities induced by MA accelerate diffusion processes.

Depending on the milling conditions, two entirely different reaction kinetics are possible [21, 54]:

- the reaction may extend to a very small volume during each collision, resulting in a* gradual* transformation, or
- if the reaction enthalpy is sufficiently high (and if the adiabatic temperature is above 1800 K), a* self-propagating high-temperature synthesis (SHS)* reaction (also known as combustion synthesis) can be initiated; by controlling the milling conditions (e.g., by adding diluents) to avoid combustion, reactions may be made to proceed in a steady-state manner.

The latter type of reactions requires a critical milling time for the combustion reaction to be initiated. If the temperature of the vial is recorded during the milling process, the temperature initially increases slowly with time. After some time, the temperature increases abruptly, suggesting that ignition has occurred, and this is followed by a relatively slow decrease.

**Figure 7:**
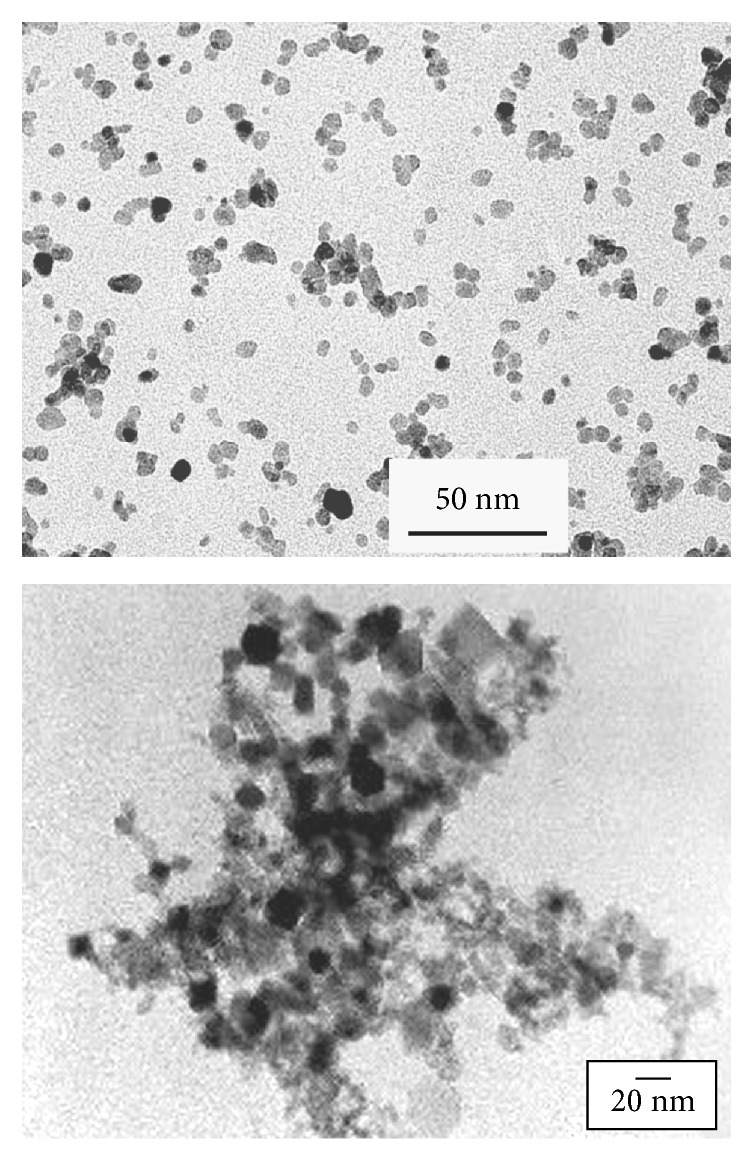
Transmission electron micrographs of ceria particles produced by MA (top) and vapor synthesis (bottom) methods. Note the severe agglomeration of the nanoparticles in the latter case.

In the above types of reactions, the milled powder consists of the pure metal* M* dispersed in the* RO* phase, both usually of nanometer dimensions. It has been shown that by an intelligent choice of the reductant, the* RO* phase can be easily leached out. Thus, by selective removal of the matrix phase by washing with appropriate solvents, it is possible to achieve well-dispersed metal nanoparticles as small as 5 nm in size. If the crystallinity of these particles is not high, it can be improved by subsequent annealing, without the fear of agglomeration due to the enclosure of nanoparticles in a solid matrix. Such nanoparticles are structurally and morphologically uniform having a narrow size distribution. This technique has been used to produce powders of many different metals—Ag, Cd, Co, Cr, Cu, Fe, Gd, Nb, Ni, Ti, V, W, Zn, and Zr. Several alloys, intermetallics, and nanocomposites have also been synthesized.  Figure 7 shows two micrographs of nanocrystalline ceria particles produced by the conventional vapor synthesis and MA methods. Even though the grain size is approximately the same in both the cases, the nanoparticles produced by MA are very discrete and display a very narrow particle size distribution, while those produced by vapor deposition are agglomerated and have a wide particle size distribution.

Novel materials have also been synthesized by MA of blended elemental powders. One of the interesting examples in this category is the synthesis of plain carbon steels and stainless steels [55, 56]. Two main advantages of this synthesis route are that the steels can be manufactured at room temperature and that the steels will be nanostructured, thus conferring improved properties. But, these powders need to be consolidated to full density for applications, and it is possible that grain growth occurs during this process. The kinetics of grain growth can, however, be controlled by regulating the time and temperature combinations.

### 7.2. Hydrogen Storage Materials

Another important application of MA powders is hydrogen storage. Mg is a lightweight metal (the lightest structural material) with a density of 1.74 g/cm^3^. Mg and Mg-based alloys can store a significant amount of hydrogen. However, being a reactive metal, Mg always contains a thin layer of oxide on the surface. This surface oxide layer, which inhibits asorption of hydrogen, needs to be broken down, through activation. A high-temperature annealing is usually done to achieve this. But, MA can overcome these difficulties due to the severe plastic deformation experienced by the powder particles during MA. A catalyst is also often required to accelerate the kinetics of hydrogen and dehydrogenation of alloys.

Mg can adsorb about 7.6 wt.% hydrogen and Mg-Ni alloys much less. However, Mg is hard to activate, its temperature of operation is high, and the sorption and desorption kinetics are slow. Therefore, Mg-Ni and other alloys are being explored. But, due to the high vapor pressure of Mg and significant difference in the melting temperatures of the constituent elements (Mg = 650°C and Ni = 1452°C), it is difficult to prepare these alloys by conventional melting and solidification processes. Therefore, MA has been used to conveniently synthesize these alloys, since this is a completely solid-state processing method. An added advantage of MA is that the product will have a nanocrystalline grain structure, allowing the improvements described below [57, 58].

**Figure 8:**
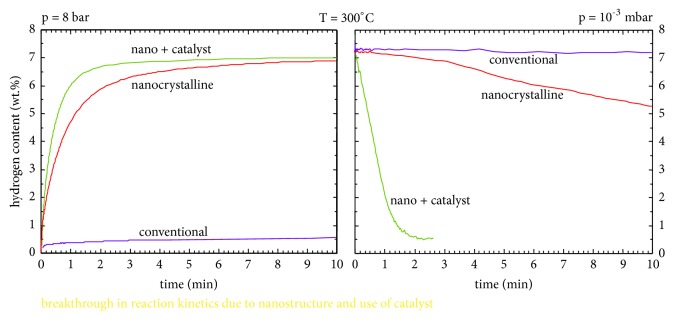
Comparison of the hydrogen sorption and desorption kinetics of Mg alloys with conventional and nanocrystalline grain sizes. Significant improvement in the desorption kinetics when a catalyst is added is worth noticing.

Figure 8 shows the hydrogenation and dehydrogenation plots for conventional and nanocrystalline alloys. An additional plot for a situation where a catalyst is added to the nanocrystalline material is also shown. From these two graphs, the following facts become clear. Firstly, the nanocrystalline alloys adsorb hydrogen much faster than conventional (coarse-grained) alloys, even though the amount of hydrogen adsorbed is the same. While the conventional alloys do not adsorb any significant amount of hydrogen in about 10 min, the allowable full amount of hydrogen is adsorbed in the nanocrystalline alloy. Similar observations are made by many other authors in many different alloys. Further, the kinetics are slightly faster (not much different) when a catalyst is added to the nanocrystalline alloy. On the other hand, the kinetics appear to be very significantly affected during the desorption process. For example, the nanocrystalline alloys desorb hydrogen much faster than the conventional alloys. Again, while the conventional alloys do not desorb any significant amount of hydrogen, the nanocrystalline alloy desorbs over 20% of the hydrogen stored in about 10 min. But, the most significant effect was observed when a catalyst was added to the nanocrystalline alloy. All the adsorbed hydrogen was desorbed in about 2 min, when a catalyst was added to the nanocrystalline alloy. Thus, nanostructure processing through MA appears to be a fruitful way of developing novel hydrogen storage materials [59].

Many novel complex hydrides based on light metals (Mg, Li, and Na) are being explored and the important categories of materials being investigated include the alanates (also known as aluminohydrides, a family of compounds consisting of hydrogen and aluminum), borohydrides, amide borohydrides, amide-imide systems (which have a high hydrogen storage capacity and low operative temperatures), amine borane (compounds of borane groups having ammonia addition), and alane.

### 7.3. Energy Materials

The technique of MA has also been employed to synthesize materials for energy applications. These include synthesis of photovoltaic (PV) materials and ultrafine particles for faster ignition and combustion purposes.

One of the most common PV materials is CIGS, a compound of Cu, In, Ga, and Se, with the composition Cu(In,Ga)Se_2_. Since PV cells have the highest efficiency when the band gap for this semiconductor is 1.25 eV, one will have to engineer the composition for the ratio of Ga to In. This is because the band gaps for CuInSe_2_ and CuGaSe_2_ are 1.02 and 1.70 eV, respectively. Accordingly, a Ga/(Ga+In) ratio of about 0.3 is found most appropriate and the final ideal composition of the PV material will be Cu_0.97_In_0.7_Ga_0.3_Se_2_.

The traditional method of synthesizing this material is to prepare an alloy of Cu, Ga, and In by vapor deposition methods and incorporate Se into this by exposure to Se atmosphere. Consequently, this is a complex process. MA, however, can be a simple, direct, and efficient method of manufacturing. Blended elemental powders of Cu, In, Ga, and Se were milled in a copper container for different periods of time, and the phase constitution was monitored through X-ray diffraction studies. It was noted that the tetragonal Cu(In,Ga)Se_2_ phase has formed even after milling the powder mix for 20 min. Process variables such as mill rotation speed, ball-to-powder weight ratio (BPR), and milling time were varied, and it was noted that the ideal composition was achieved at low milling speeds, short milling times, and a low BPR (Table 2) [60]. By fine-tuning these parameters, it should be possible to achieve the exact composition.

Nanostructured metal particles have high specific surface areas and consequently they are highly reactive and can store energy. This area has received a great impetus in recent years since the combustion rates of nanostructured metal particles are much higher than those of coarse metal particles. The particle size cannot be exceedingly small because the surface may be coated with a thin oxide layer and make the particles passive [61–63]. MA is very well suited to prepare such materials because the reactions are easily initiated by impact or friction, and at the same time, milling is a simple, scalable, and controllable technology capable of mixing reactive components on the nanoscale. These reactive materials could be used as additives to propellants, explosives, and pyrotechnics as well as manufacture of reactive structural components [63].

Compared to the presently used liquid aviation fuels, metal particles based on B, Al, and Mg are effective. However, B and Al have very high boiling points and high heats of enthalpy. On the other hand, Mg has a low boiling point, but also low heat of enthalpy. Therefore, if Al and Mg could be mixed intimately, on a nanometer scale, one could hope to have a high heat of enthalpy at reduced boiling temperatures. That is, Mg-Al alloy particles could be practical in achieving a high-energy density and practical ignition/boiling temperatures. MA is a very effective method to accomplish this.

**Figure 9:**
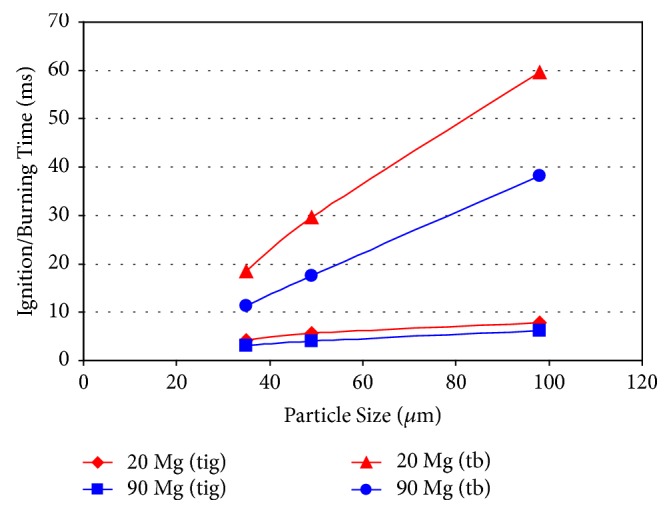
Ignition and burning times as a function of particle size for the mechanically alloyed Al-20 and Al-90 at.% Mg powders. In the insert, t_ig_ and t_b_ represent the ignition and burning times, respectively.

Al and Mg powders were blended together in different proportions and milled. Since the milled powders have a range of particle sizes, they were sieved to obtain well-defined powder sizes. They were then fluidized and then combusted in a CH_4_-air premixed flame. Both the ignition and burning times of these metal particles decreased with decreased particle sizes and were also shorter when the Mg content is higher (Figure 9) [64]. Similar results have been reported by other investigators.

## 8. Powder Consolidation

The product of MA is in powder form. Except in cases of chemical applications, such as catalysis, when the product does not require consolidation, widespread application of MA powders requires efficient methods of consolidating them into bulk shapes. Successful consolidation of MA powders is a nontrivial problem since fully dense materials should be produced while simultaneously retaining the nanometer-sized grains without coarsening or the amorphous phases without crystallizing. Conventional consolidation of powders to full density through processes such as hot extrusion [65] and hot isostatic pressing (HIP) [66] requires use of high pressures and exposure to elevated temperatures for extended periods of time [67]. Unfortunately, however, this results in significant coarsening of the nanometer-sized grains or crystallization of amorphous phases, and consequently the benefits of nanostructure processing or amorphization are lost [68]. On the other hand, retention of fine microstructures requires use of low consolidation temperatures, and then it is difficult to achieve full interparticle bonding at these low temperatures. Therefore, novel and innovative methods of consolidating MA powders are required. The objectives of consolidation of these powders are to (a) achieve full densification (without any porosity), (b) minimize microstructural coarsening (i.e., retention of fine grain size), and/or (c) avoid undesirable phase transformations. In fact, the early results of excellent mechanical properties, and most specifically superplasticity at room temperature [69], attributed to nanocrystalline ceramics have not been successfully reproduced, due to incomplete consolidation of the nanopowders in the material tested. Thus, consolidation to full density assumes even greater importance.

Because of the small size of the powder particles (typically a few micrometers, even though the grain size is only a few nanometers), MA powders pose special problems. For example, such nanometric powders are very hard and strong. They also have a high level of interparticle friction. Further, since these powders have a large surface area, they also exhibit high chemical reactivity. Therefore, special precautions need to be taken to consolidate the metal powders to bulk shapes.

Successful consolidation of MA powders has been achieved by electrodischarge compaction, plasma-activated sintering, shock (explosive) consolidation [70], HIP, hydrostatic extrusion, strained powder rolling, and sinter forging [71]. By utilizing the combination of high temperature and pressure, HIP can achieve a particular density at lower pressure when compared to cold isostatic pressing or at lower temperature when compared to sintering. Because of the increased diffusivity in nanocrystalline materials, sintering (and therefore densification) takes place at temperatures much lower than those in coarse-grained materials. This is likely to reduce the grain growth. Spark plasma sintering (SPS) has become the most popular technique to consolidate metal powders, especially those with ultrafine grain sizes due to the fact that full density can be achieved at low temperatures and shorter times, in comparison to several other methods [72, 73]. An excellent review by Groza [71] may be consulted for full details of the methods of consolidation and description of results.

## 9. Powder Contamination

A major concern in metal powders processed by MA methods is the nature and amount of impurities that get incorporated into the powder and contaminate it. The small size of the powder particles and consequent availability of large surface area, formation of fresh surfaces during milling, and wear and tear of the milling tools and the atmosphere under which the powder is milled all contribute to contamination of the powder. In addition to these factors, one has to consider the purity of the starting raw materials and the milling conditions employed (especially the milling atmosphere). Thus, it appears as though powder contamination is an inherent drawback of the method and that the milled powder will always be contaminated with impurities unless special precautions are taken to avoid/minimize them. The magnitude of powder contamination appears to depend on the time of milling, intensity of milling, atmosphere in which the powder is milled, nature and size of the milling medium, and differences in the strength/hardness of the powder and the milling medium and the container.

**Figure 10:**
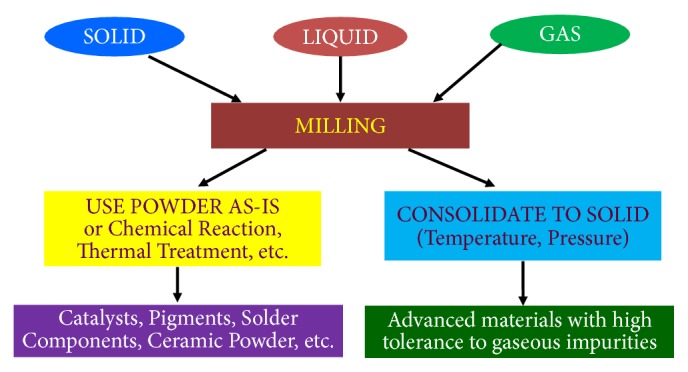
Alternatives to consolidation of metal powders. If powders are used as catalysts, pigments, solder components, etc., then they need not be consolidated. However, if powders need to be consolidated, then they could be used in applications with high tolerance to gaseous impurities.

Several attempts have been made in recent years to minimize powder contamination during milling. An important point to remember is that if the starting powders are highly pure, then the final product is going to be clean and pure, with minimum contamination from other sources, provided that proper precautions are taken during milling. It is also important to keep in mind that all applications do not require ultraclean powders and that the purity desired will depend on the specific application (Figure 10). But, it is a good practice to minimize powder contamination at every stage and ensure that additional impurities are not picked up subsequently during handling and/or consolidation.

One way of minimizing contamination from the grinding medium and the milling container is to use the same material for the container and the grinding medium as the powder being milled. Thus, one could use copper balls and copper container for milling copper and copper alloy powders [60]. If a container of the same material to be milled is not available, then a thin adherent coating on the internal surface of the container (and also on the surface of the grinding medium) with the material to be milled will minimize contamination. The idea here is to mill the powder once allowing the powder to be coated onto the grinding medium and the inner walls of the container. The milled loose powder is then discarded and a fresh batch of powder is milled, but with the old grinding media and container. In general, a simple rule that should be followed to minimize contamination from the milling container and the grinding medium is that the container and grinding medium should be harder/stronger than the powder being milled.

Milling atmosphere is perhaps the most important variable that needs to be controlled. Even though nominally pure gases are used to purge the glove box before loading and unloading the powders, even small amounts of impurities in the gases appear to contaminate the powders, especially when they are reactive. The best solution one could think of will be to place the mill inside a chamber that is evacuated and filled with high-purity argon gas. Since the whole chamber is maintained under argon gas atmosphere (continuously purified to keep oxygen and water vapor below 1 ppm each) and the container is inside the mill, which is inside the chamber, contamination from the atmosphere is minimum.

Contamination from PCAs is perhaps the most ubiquitous. Since most of the PCAs used are organic compounds, which have low melting and boiling points, they decompose during milling due to the heat generated. The decomposition products consisting of carbon, oxygen, nitrogen, and hydrogen react with the metal atoms and form undesirable carbides, oxides, nitrides, etc.

## 10. Concluding Remarks and Future Prospects

**Figure 11:**
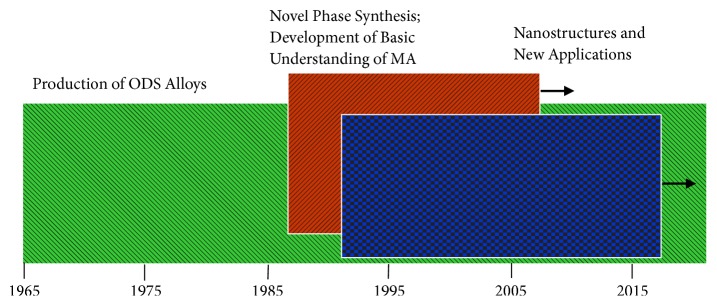
Schematic showing the different periods of development in the field of mechanical alloying (MA) and applications of MA products since its inception in 1965.

We have only briefly touched upon the scientific and technological aspects of MA materials, along with some of the concerns, such as powder contamination and consolidation issues. The historical development of MA during the last 50 years or so can be divided into three major periods (Figure 11). The first period, covering the first twenty years (from 1966 up to about 1985), was mostly concerned with the development and production of oxide-dispersion-strengthened (ODS) superalloys for applications in the aerospace industry. Several alloys, with improved properties, based on Ni and Fe were developed and found useful applications. These included the MA754, MA760, MA956, MA957, and MA6000 [74]. A more recent survey of application of MA to steel is also available [75]. The second period of about fifteen years, covering from about 1986 up to about 2000, witnessed advances in the fundamental understanding of the processes that take place during MA. Along with this, a large number of dedicated conferences were held, and there was a burgeoning of publication activity. Comparisons have also been frequently made between MA and other nonequilibrium processing techniques, more specifically RSP. It was shown that the metastable effects achieved by these two nonequilibrium processing techniques are similar. Revival of the mechanochemical processing (MCP) took place, and a variety of novel substances were synthesized. Several modeling studies were also conducted to enable prediction of the phases produced or microstructures obtained, although with limited success. The third period, starting from about 2001, saw a reemergence of the quest for new applications of the MA materials, not only as structural materials, but also for chemical applications such as catalysis and hydrogen storage, with the realization that contamination of the milled powders is the limiting factor in the widespread applications of the MA materials. Innovative techniques to consolidate the MA powders to full density while retaining the metastable phases (including glassy phases and/or nanostructures) in them were also developed. All these are continuing with the ongoing investigations to enhance the scientific understanding of the MA process.

At present, activities relating to both the science and technology of MA have been going on simultaneously. While some groups have been focusing on developing a better understanding of the process and optimizing the process variables to achieve a certain phase or phase combination in the alloy system, others have been concentrating on exploiting this technique for different applications. It has also come to be realized that the MA process can be used to recycle used and discarded materials to produce value-added materials for subsequent consumption. They can be pulverized, separated, and used to mix with other powders to produce composites. The bottom line is that newer and improved materials are being continuously developed for novel applications.

## Figures and Tables

**Table 1:** Attributes of mechanical alloying.

(1)	Production of fine dispersion of second phase (usually oxide) particles
(2)	Extension of solid solubility limits
(3)	Refinement of grain sizes down to nanometer range
(4)	Synthesis of novel crystalline and quasicrystalline phases
(5)	Development of amorphous (glassy) phases
(6)	Disordering of ordered intermetallics
(7)	Possibility of alloying of difficult to alloy elements/metals
(8)	Inducement of chemical (displacement) reactions at low temperatures for (a) Mineral and Waste processing, (b) Metals refining, (c) Combustion reactions, and (d) Production of discrete ultrafine particles
(9)	Scaleable process

**Table 2:** Chemical analysis (at.%) of the Cu-In-Ga-Se powder mechanically alloyed under different processing conditions.

BPR	Speed (rpm)	Time (min)	Cu	In	Ga	Se
20:1	300	120	32.61	15.23	6.53	45.62

20:1	300	240	31.73	15.63	6.71	45.93

10:1	150	40	25.98	16.98	7.25	49.78

Targeted (Cu_0.97_In_0.7_Ga_0.3_Se_2_)			24.43	17.63	7.56	50.38
